# Recruitment Challenges in Mother–Infant Research: Factors Associated With Low Enrollment and Inclusion in the Synbio‐Breast Study

**DOI:** 10.1155/bmri/2358274

**Published:** 2026-07-24

**Authors:** Sandra Simons, Hans van Goudoever, Berber Vlieg-Boerstra

**Affiliations:** ^1^ Department of Pediatrics, OLVG Hospital, Amsterdam, the Netherlands; ^2^ Department of Pediatrics, Emma Children′s Hospital, Amsterdam UMC, University of Amsterdam, Amsterdam, the Netherlands, uva.nl

**Keywords:** human milk, maternal diet, microbiome, participation rates, recruitment, Synbio-Breast study

## Abstract

**Background:**

An optimal early‐life environment is crucial for child health, with human milk and the infant microbiome playing central roles. The Synbio‐Breast study investigates how maternal diet contributes to the synbiotic composition of human milk in atopic women. Despite increasing societal interest in this topic, recruitment proved challenging.

**Objective:**

The aim of this study is to quantify and analyze factors associated with low enrollment and inclusion rates and to explore strategies for improving recruitment in future mother–infant studies.

**Methods:**

Enrollment and inclusion rates and temporal trends were assessed using linear regression models, whereas associations between facilitating factors—including involvement of the PhD student, recruitment strategy, lactation meetings, and attendance at the Nine Months Fair—and enrollment were assessed using logistic regression models.

**Results:**

Of the 681 women screened, 215 expressed willingness to participate. Despite multiple efforts and adaptations to enhance engagement, enrollment remained low. After adjusting for the COVID‐19 period, only recruitment conducted by the PhD student via email—without personal contact through a healthcare professional—was significantly associated with lower odds of enrollment. Of the 215 enrolled participants, 75 were ultimately included (11% of all screened women). Key reasons for exclusion were partial breastfeeding (30.0%), antibiotic use (22.1%), and loss of interest (19.3%).

**Conclusion:**

Strict inclusion and exclusion criteria contributed to substantial low enrollment and inclusion rates in the Synbio‐Breast study. Successful recruitment in mother–infant research requires more than logistical organization; it depends on personalized engagement built on existing relationships of trust. Moreover, studies that offer no direct health benefit to participants—such as the Synbio‐Breast study—tend to have inherently lower uptake, underscoring the need for tailored and relationship‐based recruitment strategies.

**Key Message**

Strict inclusion and exclusion criteria, combined with limited personal contact during recruitment, substantially reduced enrollment and inclusion rates in the Synbio‐Breast study. Strengthening trusted relationships using personalized, face‐to‐face approaches and providing personal benefits are essential to improving recruitment in future mother–infant research.

## 1. Introduction

Participant recruitment remains one of the most critical challenges in clinical research, directly affecting study feasibility, statistical power, and external validity [1–3]. Across disciplines, insufficient recruitment is a major determinant of trial failure, with many studies failing to meet enrollment targets, requiring protocol amendments, or being prematurely discontinued due to low accrual rates [4, 5]. Despite its importance, recruitment processes and barriers are often underreported, limiting the ability to generalize lessons learned across studies [6].

Recruitment challenges may be particularly pronounced in studies involving healthy or low‐risk populations, where perceived personal benefit is limited and participation relies primarily on individual motivation rather than clinical necessity [7]. In such contexts, participation is less driven by medical need and more by behavioral and contextual factors, including perceived relevance of the study, trust in the recruiter, and the mode of communication [6, 7].

In mother–infant research, additional complexities arise due to the burden of participation during pregnancy and the early postpartum period, competing priorities, and the practical and emotional demands associated with this life phase. These factors may further reduce willingness to participate, particularly in studies that do not offer direct clinical benefit.

The Synbio‐Breast study, initiated in April 2018, was designed to investigate the association between maternal diet and the synbiotic composition of human milk in atopic lactating women and healthy lactating controls. The synbiotic composition refers to the composition of microbes, human milk oligosaccharides (HMOs), and short‐chain fatty acids (SCFAs). In a follow‐up study, these outcomes will be related to allergy prevention in infants. To minimize confounding, stringent inclusion and exclusion criteria were applied [8, 9]. Although scientifically relevant, the study offered no direct clinical benefit to participants, thereby relying heavily on voluntary engagement [7].

Despite an initially estimated recruitment period of 2.5 years, the inclusion period ultimately extended to 7 years, with completion in September 2025. This substantial delay highlights the need to better understand the drivers of recruitment success and failure in this context. Importantly, design choices intended to strengthen internal validity—such as strict eligibility criteria—may have inadvertently reduced recruitment feasibility [9].

The primary aim of this study was therefore to quantify recruitment efficiency and identify factors associated with enrollment and inclusion. Specifically, we evaluated temporal trends in recruitment, assessed the impact of different recruitment strategies, and explored how contextual and behavioral factors influenced willingness to participate. By doing so, we aim to provide practical insights to improve recruitment strategies in future mother–infant research.

## 2. Study Design

The sample size was based on the ability to detect an association between dietary intake of food‐derived microbes and their levels in human milk using multivariable linear regression.

Because no directly comparable observational data were available, the study by Mastromarino et al. [10] was considered only as contextual evidence that measurable changes in human milk microbial composition are biologically plausible. Given differences in study design (interventional vs. observational) and outcome reporting, this information should be interpreted as approximate.

A moderate effect size was assumed, represented by a partial correlation of approximately 0.35 between exposure and outcome. This value lies within the range typically considered a moderate correlation (approximately 0.3–0.4) according to conventional benchmarks [11]. Using a two‐sided *α* of 0.05 and 80% power, this yielded an estimated minimum sample size of 65 participants in both correlation‐based and multivariable regression sample size calculations. Given the exploratory and hypothesis‐generating nature of the study, the sample size should be interpreted as sufficient to detect moderate associations rather than small effects.

## 3. Methods

Pregnant women attending the outpatient clinic of gynecology and obstetrics at OLVG or affiliated midwifery practices were prescreened during pregnancy for eligibility based on atopic status and predefined inclusion and exclusion criteria (Table 1). If they met the inclusion criteria, they were invited to participate in the Synbio‐Breast study. Participants received written patient information and informed consent was obtained after a minimum reflection period of 14 days. Participants received a reimbursement of €25 for participation.

**Table 1:** Inclusion and exclusion criteria for participation in the Synbio‐Breast study.

Inclusion criteria	Exclusion criteria
Vaginal delivery	Cesarean section, premature delivery (< 37 weeks gestation)
Intention to breastfeed for at least 1 month	More than two supplementary bottle feedings in the first month
Pre‐pregnancy BMI < 35 kg/m^2^	Use of antibiotics within 3 months before or during study period
	Use of probiotics within 4 weeks before or during study period
Atopic disease (e.g., hay fever, house dust mite allergy, food allergies, animal allergies, atopic dermatitis, and asthma)	Diabetes (including gestational diabetes) and gastrointestinal disorders

Initial recruitment efforts included direct approach during outpatient visits, distribution of flyers, social media outreach, and attendance at local events such as lactation meetings and the “Nine Months Fair.” Additional recruitment strategies were implemented over time, including outreach through personal and professional networks, schools, maternity care services, lactation consultants, and other hospitals, disseminating calls via social media, prenatal courses, pregnancy forums, and allergy‐related online communities.

As this study follows an observational research design, reporting of participant selection and baseline characteristics would conventionally align with the STROBE (Strengthening the Reporting of Observational Studies in Epidemiology) criteria, the standard guideline for observational cohort and other nonrandomized clinical studies [12]. However, due to the implementation of two sequential inclusion checkpoints, the participant selection process could be more clearly represented by the staged eligibility‐to‐enrollment flow typically described in randomized controlled trials. To ensure transparent and standardized reporting of participant progression across these sequential inclusion phases, the CONSORT (Consolidated Standards of Reporting Trials) framework was applied to describe and quantify participant flow [13].

Accordingly, two key study rates were defined in line with CONSORT participant flow principles: the enrollment rate, calculated as the proportion of participants enrolled among those eligible (enrolled/eligible), and the inclusion rate, defined as the proportion of participants included in the final study cohort among those enrolled (included/enrolled).

Monthly enrollment and inclusion rates were calculated and expressed as percentages. Temporal trends in these rates were analyzed using linear regression models based on monthly aggregated data. Time (in months since study start) was included as a continuous variable, and a binary variable representing the COVID‐19 period (February 2020 to February 2022) was included to estimate its additional effect on enrollment and inclusion rates.

Model assumptions were assessed by visual inspection of residual plots, and residual independence was evaluated using autocorrelation plots.

Associations between facilitating factors and enrollment were analyzed using logistic regression models restricted to eligible participants. The outcome variable was enrollment (yes vs. no), and results are presented as odds ratios (ORs) with 95% confidence intervals. Analyses were performed both unadjusted and adjusted for the COVID‐19 period, given the known impact of the COVID‐19 pandemic on participant recruitment in non‐COVID‐19 clinical research [13, 14].

Statistical significance was defined as a two‐sided *p* value < 0.05.

## 4. Results

**Figure 1:**
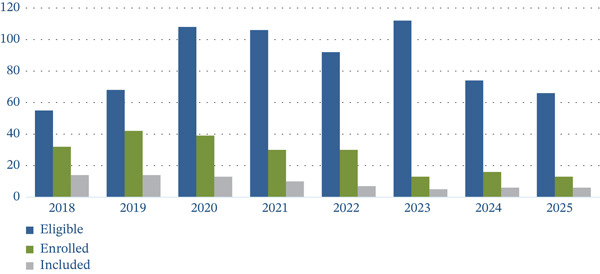
Numbers of eligible, enrolled, and included women per year.

Between April 2018 and August 2025, 681 prescreened women were found eligible for participation in the Synbio‐Breast study. Of these, 215 women were enrolled antenatally (31.6% enrollment rate). After delivery, when all inclusion and exclusion criteria could be evaluated, 75 of the 215 women were included in the study, corresponding to a 34.9% inclusion rate. Overall, 75 of the 681 eligible women (11%) were included. The distribution of eligible, enrolled, and included participants is presented in Figure 1.

Temporal trends in enrollment and inclusion rates were analyzed using linear regression models based on monthly aggregated data. Enrollment and inclusion rates were calculated per month and expressed as percentages. Time (in months since study start) was included as a continuous independent variable.

The estimated regression coefficient represents the average change in percentage points per month. A significant negative time trend in enrollment rate was observed in the unadjusted model, with a decrease of 0.57 percentage points per month (unstandardized regression coefficient; 95% CI −0.79 to −0.34; *p* < 0.001). No significant time trend was observed for inclusion rate (−0.02 percentage points per month; 95% CI −0.32 to 0.37; *p* = 0.889).

**Figure 2:**
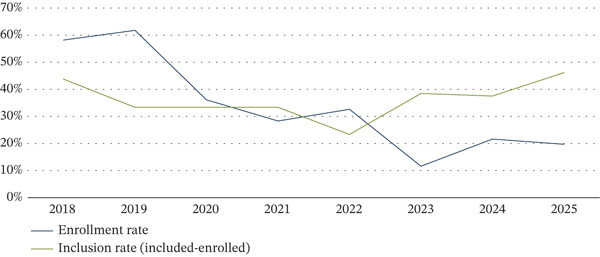
Time trends in enrollment rates (enrolled–eligible) and inclusion rates (included–enrolled). Rates were calculated monthly; the *x*‐axis is displayed in calendar years for readability.

These trends are visualized in Figure 2. The *x*‐axis is displayed in calendar years for readability, whereas the underlying model was fitted using time expressed in months.

In a model including both time and the COVID‐19 period, the time coefficient for enrollment was −0.65 percentage points per month (95% CI −0.87 to −0.43; *p* < 0.001), indicating a continued negative trend over time. The COVID‐19 period was associated with a 19.9 percentage point lower enrollment rate (95% CI −31.8 to −8.04; *p* = 0.001). No significant time trend was observed for inclusion rate after adjustment (−0.01 percentage points per month; 95% CI −0.36 to 0.34; *p* = 0.945), and no effect of the COVID‐19 period was found (−11.4 percentage points; 95% CI −30.9 to 8.16; *p* = 0.250).

Of the 215 enrolled women, 140 (65.1%) were excluded. The main reasons for exclusion after enrollment were formula feeding (> 2 bottle feedings), antibiotic use during labor or postpartum, withdrawal of interest in participation, or cesarean section. Table 2 shows an overview of all the reasons for exclusion after enrollment.

**Table 2:** Reasons for exclusion after enrollment (*N* = 140).

Reason for exclusion	*N*(%)
Formula feeding	42 (30.0%)
Antibiotic use	31 (22.1%)
Loss of interest	27 (19.3%)
Cesarean section	17 (12.1%)
Not responding	9 (6.4%)
Probiotic use	4 (2.9%)
No atopic disease (screening failure)	4 (2.9%)
Development of diabetes gravidarum	2 (1.4%)
Unknown	2 (1.4%)
Birth weight < p3	1 (0.7%)
Birth weight > p97	1 (0.7%)

To better understand potential influences on willingness to participate, we examined a set of facilitating factors that were hypothesized to affect enrollment: the appointment of a dedicated PhD researcher during the recruitment period, the discontinuation of referrals through healthcare providers in favor of direct recruitment by the PhD researcher, face‐to‐face recruitment at lactation meetings, and recruitment during the “Nine Months Fair.” Associations between these facilitating factors and enrollment were analyzed using logistic regression models, restricted to eligible participants. The outcome variable was enrollment (yes vs. no), and results are presented as ORs with 95% confidence intervals (Table 3).

**Table 3:** Association between facilitating factors and enrollment: unadjusted and COVID‐19–adjusted logistic regression analyses.

Facilitating factor	Unadjusted odds ratios	Adjusted odds ratios
PhD student appointment	**OR 0.26**	OR 1.07
	**(95% CI 0.15–0.42)**	(95% CI 0.52–2.18)
	**p** < 0.001	*p* = 0.861
Direct through PhD student (e‐mail)	**OR 0.30**	**OR 0.38**
	**(95% CI 0.20–0.43)**	**(95% CI 0.19–0.76)**
	**p** < 0.001	**p** = 0.006
Lactation meetings	**OR 2.99**	OR 1.41
	**(95% CI 1.43–6.40)**	(95% CI 0.65–3.12)
	**p** = 0.004	*p* = 0.387
Nine Months Fair	OR 0.96	OR 1.25
	(95% CI 0.26–2.99)	(95% CI 0.33–3.94)
	*p* = 0.950	*p* = 0.713

*Note:* The bolded values indicate that statistical significance was defined as a two‐sided p value < 0.05.

In the unadjusted analyses, the presence of the PhD researcher was associated with lower odds of enrollment (OR 0.26, 95% CI 0.15–0.42, p < 0.001). Direct recruitment by the PhD researcher was also associated with lower odds of enrollment (OR 0.30, 95% CI 0.20–0.43, *p* < 0.001). In contrast, recruitment through lactation meetings was associated with higher odds of enrollment (OR 2.99, 95% CI 1.43–6.40, *p* = 0.004), whereas recruitment during the Nine Months Fair showed no significant association with the odds of enrollment.

After adjustment for the COVID‐19 period, most associations were attenuated. The presence of the PhD researcher was no longer significantly associated with enrollment (OR 1.07, 95% CI 0.52–2.18, *p* = 0.861). In contrast, direct recruitment by the PhD researcher remained significantly associated with lower odds of enrollment (OR 0.38, 95% CI 0.19–0.76, *p* = 0.006). None of the other facilitating factors, including recruitment through lactation meetings or the Nine Months Fair, showed statistically significant associations after adjustment.

## 5. Discussion

Understanding the factors that influenced recruitment is essential for interpreting the feasibility and generalizability of our findings. Although the strict inclusion and exclusion criteria shaped the composition of the study population, the observed variation in recruitment rates appeared to have arisen from more subtle and context‐dependent dynamics.

In this study, we found that variation in the enrollment rate appeared to be influenced less by clinical factors and more by women′s motivation and the context of recruitment. The four facilitating factors examined in this study illustrate how subtle differences in recruitment approach may influence enrollment. We examined (1) whether the PhD student was involved in recruitment, (2) whether contact was made indirectly via caregivers or directly by the PhD student, (3) recruitment during lactation meetings, and (4) recruitment during the Nine Months Fair. In the unadjusted analyses, enrollment was lower when the PhD student was involved and when direct contact occurred via email, whereas enrollment was higher during lactation meetings. After adjustment for the COVID‐19 period, only email‐based contact by the PhD student—as opposed to direct, face‐to‐face contact by the regular healthcare provider—remained significantly associated with lower enrollment.

These differences may be explained by the recruitment strategies used and the biases they introduced [14–16]. Recruitment by healthcare providers fostered trust through existing relationships but led to selection bias, as they tended to approach women perceived as motivated. When the PhD student later contacted all eligible women directly, the recruitment pool became more representative, including women less inclined to participate, which lowered enrollment without reflecting reduced motivation. Direct face‐to‐face contact via caregivers could have helped maintain participant engagement, but this approach was used inconsistently. Email contact, on the other hand, was consistent but less personal and easily overlooked, reducing engagement. Recruitment during lactation meetings likely reflects self‐selection bias, as only self‐presenting, breastfeeding‐motivated women were recorded, whereas recruitment at the Nine Months Fair did not meaningfully influence participation.

An additional consideration is that the underlying motivation to participate in this study may have been limited. Despite sustained recruitment efforts over more than 7 years—substantially longer than the initially planned 2.5 years—recruitment remained challenging. We hypothesize that, as the study offered no direct health benefit for either mother or child, this may have reduced willingness to enroll. Although participant motivation was not formally assessed, this interpretation aligns with findings from Rodger et al. [7], who reported that 68% of women in their study participated due to anticipated health benefits, whereas only 5% cited altruistic reasons. This suggests that studies offering no immediate personal benefit face inherent barriers to participation, even when logistical and procedural factors are optimized.

Building on this, citizen science may offer alternative approaches to enhancing participant engagement [17, 18]. In citizen science projects, members of the public actively contribute to one or more stages of the research process, thereby strengthening the connection between scientific aims and participants′ own interests. Such initiatives have been shown to improve participation by fostering a sense of ownership, transparency, and societal relevance. Integrating elements of citizen science—such as providing individual feedback or enabling participants to engage more directly with the study′s goals—could therefore represent a promising strategy to increase inclusion in future research.

Relatedly, the manner in which the study was presented to potential participants also warrants consideration. The Belgian Isala project demonstrated that, apart from personal benefit, clear communication of personal relevance could markedly increase participation [19]. By emphasizing the potential health implications of the vaginal microbiome and providing participants with individual microbiome feedback, Isala recruited nearly 6000 women despite an initial target of 200. This example illustrates that a more targeted and participant‐oriented communication strategy might improve enrollment and inclusion.

## 6. Conclusion

In this study, we found that the low inclusion rate was partly driven by strict inclusion and exclusion criteria. Although all women approached for participation met the antenatal eligibility criteria during prescreening, approximately 65% of those enrolled were later excluded or withdrew. This high exclusion rate may appear disadvantageous from a feasibility perspective and caused a considerable delay. However, it resulted in a clinically homogeneous study population, reducing potential confounding and thereby strengthening internal validity.

Beyond these methodological constraints, low enrollment and inclusion rates were further influenced by women′s willingness to take part in the study. The absence of direct health benefits for the mother or infant likely contributed to reduced motivation to participate. Moreover, our findings demonstrate that personal engagement with healthcare professionals or researchers is essential: face‐to‐face contact enables researchers to build trust, communicate the value and purpose of the study, and foster a sense of shared commitment.

Experiences from other large‐scale women′s health projects show that participation can improve markedly when research is communicated in a way that emphasizes its personal relevance and offers participants meaningful insight into their own data. Approaches that strengthen the perceived value of participation have proven effective in comparable contexts. These strategies indicate that enhancing involvement and perceived benefit may be key to improving inclusion.

In studies where the benefit is collective rather than individual, recruitment success is therefore not merely a logistical challenge, but a relational one. Active, empathetic, and direct interaction with potential participants—combined with clear communication of personal relevance—appears not optional but essential for generating willingness to participate and achieving meaningful inclusion.

### 6.1. Recommendations to Boost Participation Rates

The recommendations to boost participation rates are listed as follows:

1. Use direct, personalized face‐to‐face contact with potential participants; leverage existing trust relationships with healthcare providers to facilitate participation.
2. Provide meaningful feedback or data insights to participants to enhance perceived value (citizen‐science approach).
3. Emphasize collective study benefits alongside indirect personal value, with targeted, participant‐oriented communication.

## Author Contributions

Sandra Simons: conceptualization (lead), formal analysis (lead), project administration (lead), writing—original draft (lead), and writing—review and editing (equal). Berber Vlieg‐Boerstra: supervision (lead) and writing—review and editing (equal). Hans van Goudoever: supervision (supporting) and writing—review and editing (supporting).

## Funding

This study was supported by the OLVG research (WO 17.186).

## Disclosure

The authors take full responsibility for the accuracy and integrity of the manuscript.

## Ethics Statement

For this study, medical ethical approval was obtained from the Medical Review Board of OLVG, No. WO 17.186.

## Conflicts of Interest

The authors declare no conflicts of interest.

## Data Availability

The data that support the findings of this study are available from the corresponding author upon reasonable request.
